# Prevalence of Primary Angle Closure Glaucoma in the Last 20 Years: A Meta-Analysis and Systematic Review

**DOI:** 10.3389/fmed.2020.624179

**Published:** 2021-01-18

**Authors:** Nan Zhang, Jiaxing Wang, Biyue Chen, Ying Li, Bing Jiang

**Affiliations:** ^1^Department of Ophthalmology of Second Xiangya Hospital, Central South University, Changsha, China; ^2^Department of Ophthalmology, School of Medicine, Emory University, Atlanta, GA, United States; ^3^Hunan Clinical Research Center of Ophthalmic Disease, Changsha, China

**Keywords:** glaucoma, prevalence, PACG, risk factor, age, gender, Asia

## Abstract

**Purpose:** This meta-analysis aims to investigate the worldwide prevalence of primary angle-closure glaucoma (PACG) and its risk factors in the last 20 years.

**Methods:** We conducted a systematic review and meta-analysis of 37 population-based studies and 144,354 subjects. PubMed, Embase, and Web of Science databases were searched for cross-sectional or cohort studies published in the last 20 years (2000–2020) that reported the prevalence of PACG. The prevalence of PACG was analyzed according to various risk factors. A random-effects model was used for the meta-analysis.

**Results:** The global pooled prevalence of PACG was 0.6% [95% confidence interval (CI) = 0.5–0.8%] for the last 20 years. The prevalence of PACG increases with age. Men are found less likely to have PACG than women (risk ratio = 0.71, 95% CI = 0.53–0.93, *p* < 0.01). Asia is found to have the highest prevalence of PACG (0.7%, 95% CI = 0.6–1.0%). The current estimated population with PACG is 17.14 million (95% CI = 14.28–22.85) for people older than 40 years old worldwide, with 12.30 million (95% CI = 10.54–17.57) in Asia. It is estimated that by 2050, the global population with PACG will be 26.26 million, with 18.47 million in Asia.

**Conclusion:** PACG affects more than 17 million people worldwide, especially leading a huge burden to Asia. The prevalence of PACG varies widely across different ages, sex, and population geographic variation. Asian, female sex, and age are risk factors of PACG.

## Introduction

Glaucoma is one of the leading causes of irreversible blindness worldwide (1). It is defined as a group of optic neuropathies associated with characteristic structural changes at the optic nerve head that cause the death of retinal ganglion cells and their axons, leading to visual field loss and blindness (2, 3). In contrast to primary open-angle glaucoma, the most common type of glaucoma, primary angle-closure glaucoma (PACG), is associated with the closure of the anterior chamber angle of the eye and is known to have a greater propensity of bilateral blindness, which lead to a huge burden to families and the society (4, 5).

In 2013, the worldwide prevalence of PACG was reported to be 0.5% [95% confidence interval (CI) = 0.11–1.36%] (6). It was also estimated that the global population with PACG would be 23.36 million in 2020 and 32.04 million in 2040, in which Asia accounts for more than three-quarters of PACG population (6). PACG has been associated with many risk factors, including ethnicity, age, and sex (6–8), and they all contribute to the prevalence. Updates in study designs and diagnostic methods of PACG alter the estimations of prevalence and population, whereas the International Society for Geographical and Epidemiological Ophthalmology (ISGEO) provides a standard PACG definition for survey (9). Prevalence of PACG varies across different ethnicities and geographical regions (10). With the rapid increase in global population and aging trends, it is critical to pool PACG prevalence and estimate up-to-date and accurate PACG prevalence, providing evidence for a future health-care plan. Besides, there have been increasing surveys of PACG with a large number of participants in recent years across the world, especially in Asia and Africa. In this study, we aimed to estimate the detailed prevalence of PACG globally in a risk factor-specific manner for the last two decades.

## Methods

The study was conducted following the Preferred Reporting Items for guidelines of Systemic Reviews and Meta-Analysis guidelines (11, 12).

### Eligibility Criteria

Studies published between January 2000 to September 2020 were included in this meta-analysis when they met the following inclusion criteria: (1) Population-based cross-sectional or cohort studies in which the prevalence of PACG from a defined geographic region was provided; (2) Studies with a clear definition of random or clustered sampling procedure; (3) PACG defined by using ISGEO (9) criteria or similar to ISGEO that based on structure and/or functional evidence of glaucomatous optic neuropathy with occludable anterior chamber angle; (4) Studies that prevalence data for PACG can be extracted or calculated. Exclusion criteria included: (1) Self-reported diagnosis of glaucoma included; (2) Non-English articles; (3) Articles using repeated data from the author's previous publications.

### Search Strategy

We conducted a systematic and comprehensive search in three electronic databases (PubMed, Embase, and Web of Science) from August to September 2020. A combination of keywords related to PACG (glaucoma, PACG, and primary angle-closure glaucoma) and epidemiology (prevalence, population, and survey) was used to identify all published papers, abstracts, and letters between January 2000 and September 2020. Besides, a hand search was used to identify target articles from the other reference list. The detailed search strategy of different databases was provided in Supplementary Table 1.

### Data Extraction

Two reviewers (NZ and BC) conducted data extraction independently based on inclusion and exclusion criteria; disagreements received final consensus after several full discussions between reviewers. Full data extraction in the data extraction sheet was completed after reviewers independently identified cases from every targeted article and reached final agreement. The following data were extracted and reported for each study: first author, year of publication, sex, age, continent, country, habitation area (urban or rural), numbers of cases, sample size, prevalence with 95% CI, and response rate (Supplementary Table 2).

### Statistical Analysis

Data are presented as prevalence (95%CIs). Forest plots were performed using the software R version 3.6.3 (R Foundation for Statistical Computing, Vienna, Austria) and the R package “meta” 13. We selected the prevalence of PACG as the main outcome. The relative risk ratios and 95% CIs of the results were compared. Heterogeneity between studies was assessed using the *I*^2^ statistic (13–16). Due to the high likelihood of heterogeneity among the selected studies, we used a random-effects model to evaluate pooled effects. Publication bias was calculated using the Funnel plots (17, 18), P-curve analysis (19), and Egger test (17) (*p* < 0.05 was considered as significant publication bias). Detailed bias for each study was described in Supplementary Tables 3, 4.

The *p*-value for prevalence difference among groups for age, sex, continent, habitation area, and decades was calculated using “metaprop” from R package “meta,” random-effects model. The *p*-value for prevalence difference among groups for sex was calculated using “metabin” from R package “meta,” random-effects model. A meta-regression test was performed for subgroup analysis, with the first category of each subgroup used as intercept. The statistical output includes a test of whether the intercept differs significantly from zero and whether other groups differ from the intercept. A value of *p* < 0.05 was considered statistically significant.

The number of people older than 40 years old with PACG was estimated by different continents. The population projection data were from the latest data of the World Population Prospects of the United Nations (20), which consisted of the latest results of national population consensus and demographic surveys from countries worldwide and also consider mortality rate and fertility rate in its projection of world population number. The estimated numbers of PACG population were calculated by multiplying the age- and region-specific prevalence from our random-effects model and the corresponding population number. Age- and region-specific prevalence were assumed to be consistent in the next 30 years' projection, as no significant difference has been found between the prevalence of last two decades by the random-effects model (*Q* = 0.22, df = 1, *p* = 0.64).

### Risk of Bias Assessment

Articles included in the study were assessed for risk of bias using two domains of the Quality in Prognosis Studies tool (21) that are relevant to observational studies (study participation and outcome measurement) (22). Appraisal of each domain provides a subjective assessment of the risk of bias (ranked as low, moderate, or high). A summary of the areas considered in the assessment of each domain is included in Supplementary Tables 3, 4.

## Results

### Search Results

**Figure 1:**
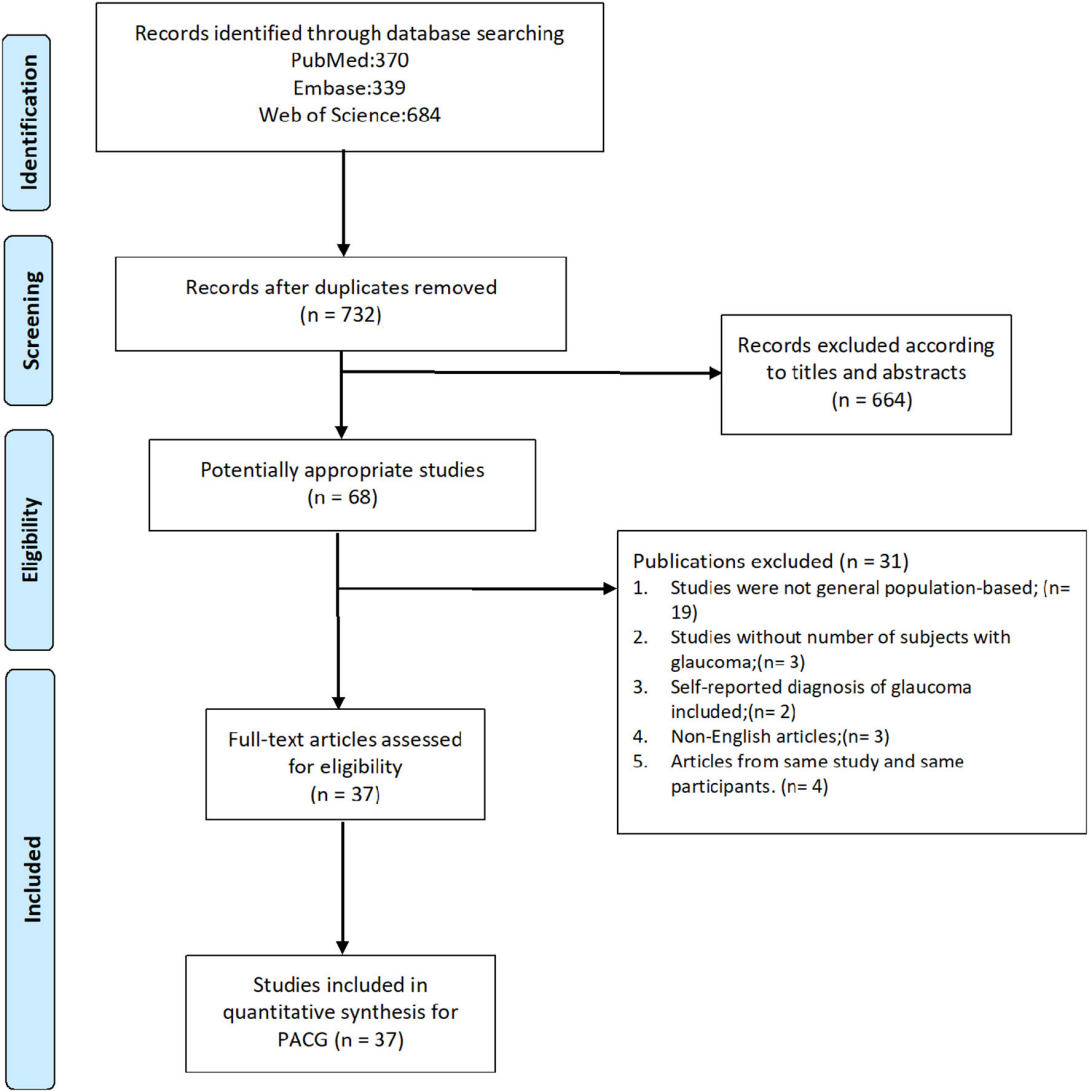
Flow charts of search process. PACG, primary angle-closure glaucoma.

In this study, we reviewed the full text of 68 studies about PACG prevalence published in the last 20 years, and 31 were excluded based on inclusion and exclusion criteria. The screening process is detailed in Figure 1. A total of 37 publications (23–59) that include 144,354 subjects were recruited. The sample size of the study ranged from 790 (Bourne, 2003, Thailand) (27) to 15,122 (Chassis, 2018, Israel) (56). Detailed information is provided in forest plots given different risk factors and summarized in Supplementary Table 2, including author, year of publication, country, continent, age range, detailed number of cases and sample size, and response rate.

### Risk of Bias

A summary of the risk of bias of the included articles is provided in Supplementary Figures 1, 2; a justification of each rating is provided in Supplementary Tables 3, 4.

Egger test result revealed a significant publication bias (*p* < 0.01) in this meta-analysis. Funnel plots and P-curve analysis results are shown in Supplementary Figures 3, 4.

### Meta-Analysis

**Figure 2:**
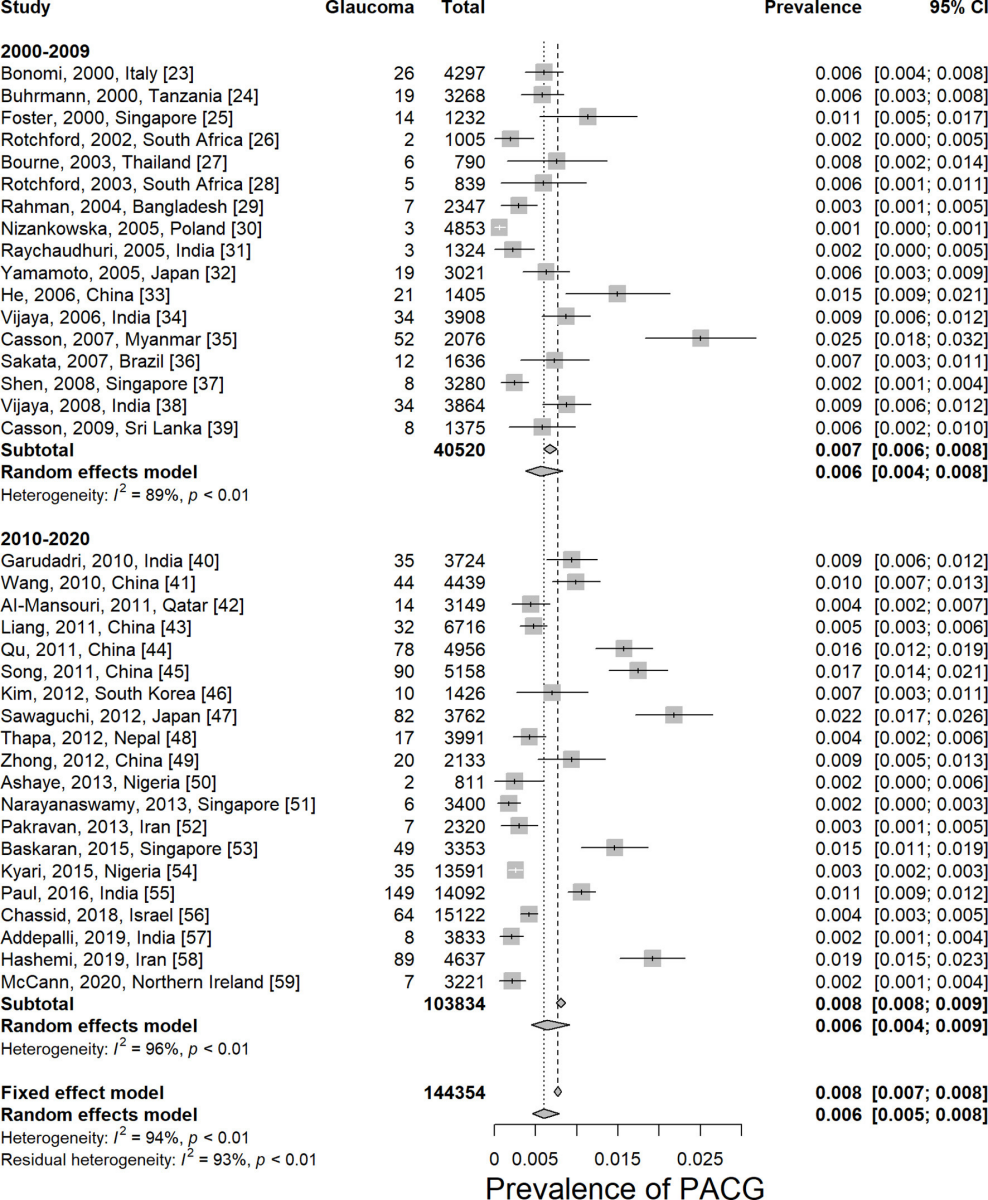
Prevalence of primary angle-closure glaucoma worldwide by decades. PACG, primary angle-closure glaucoma.

The prevalence of PACG is provided in Table 1. The overall PACG pooled prevalence worldwide is 0.6% (95% CI = 0.5–0.8%) for the last 20 years (Figure 2).

**Table 1:** Results of subgroup analyses and meta-regression analyses based on age, sex, geographical location, habitation area, decades, and risk of bias.

	**Subgroup analysis**			**Meta-regression**	
	**Number of estimates**	**Pooled estimate (95% CIs)**	***I*^**2**^, %**	**Mean difference (95% CIs)**	***P*-value**
**All estimates**	37	0.6 (0.5–0.8)	94.4		
**Age range, years**				Intercept = “<40”	
<40	1	0.2 (0–0.6)	-	−6.56 (−8.63 to −4.49)	<0.01
40–49	18	0.1 (0–0.3)	88.6	0.18 (−1.95 to 2.31)	0.87
50–59	21	0.5 (0.3–0.8)	85.8	1.26 (−0.85 to 3.37)	0.24
60–69	21	1.0 (0.7–1.5)	81.4	1.97 (−0.14 to 4.07)	0.07
70–79	11	1.4 (0.9–2.3)	73.2	2.25 (0.11 to 4.39)	0.04
70+	9	2.1 (1.2–3.3)	88.0	2.65 (0.50 to 4.79)	0.02
80+	10	2.8 (1.7–4.7)	25.3	2.79 (0.61 to 4.96)	0.01
**Sex**				Intercept = Male	
Male	26	0.8 (0.6–1.1)	90.5	−5.24 (−5.60 to −4.89)	<0.01
Female	26	0.5 (0.3–0.8)	90.8	−0.41 (−0.07 to 0.90)	0.09
**Geographical location**				Intercept = Africa	
Africa	5	0.4 (0.2–0.5)	46.3	−5.69 (−6.39 to −4.99)	<0.01
Asia	28	0.7 (0.6–1.0)	94.3	0.78 (0.04 to 1.53)	0.04
Europe	3	0.2 (0.1–0.6)	85.4	−0.42 (−1.53 to 0.68)	0.45
S. America	1	0.7 (0.4–1.3)	-	0.75 (−0.85 to 2.34)	0.36
**Habitation area**				Intercept = Urban	
Urban	9	0.7 (0.5–1.2)	90.6	−4.90 (−5.39 to −4.40)	<0.01
Rural	15	0.8 (0.5–1.2)	94.4	0.06 (−0.56 to 0.69)	0.84
Mixed or unknown	17	0.5 (0.3–0.7)	93.6	−0.39 (−1.01 to 0.22)	0.21
**Decades**				Intercept = 2000–2009	
2000–2009	17	0.6 (0.4–0.8)	89.5	−5.18 (−5.57 to −4.78)	<0.01
2010–2019	20	0.6 (0.4–0.9)	95.9	0.13 (−0.40 to 0.66)	0.63
**Risk of bias**				Intercept = Low	
Low	21	0.7 (0.5–1.0)	95.4	−4.97 (−5.31 to −4.63)	<0.01
Moderate	15	0.5 (0.3–0.7)	90.0	−0.36 (−0.88 to 0.17)	0.18
High	1	0.1 (0.7–1.3)	–	0.35 (−1.16 to 1.87)	0.65

*S. America, South America*.

**Figure 3:**
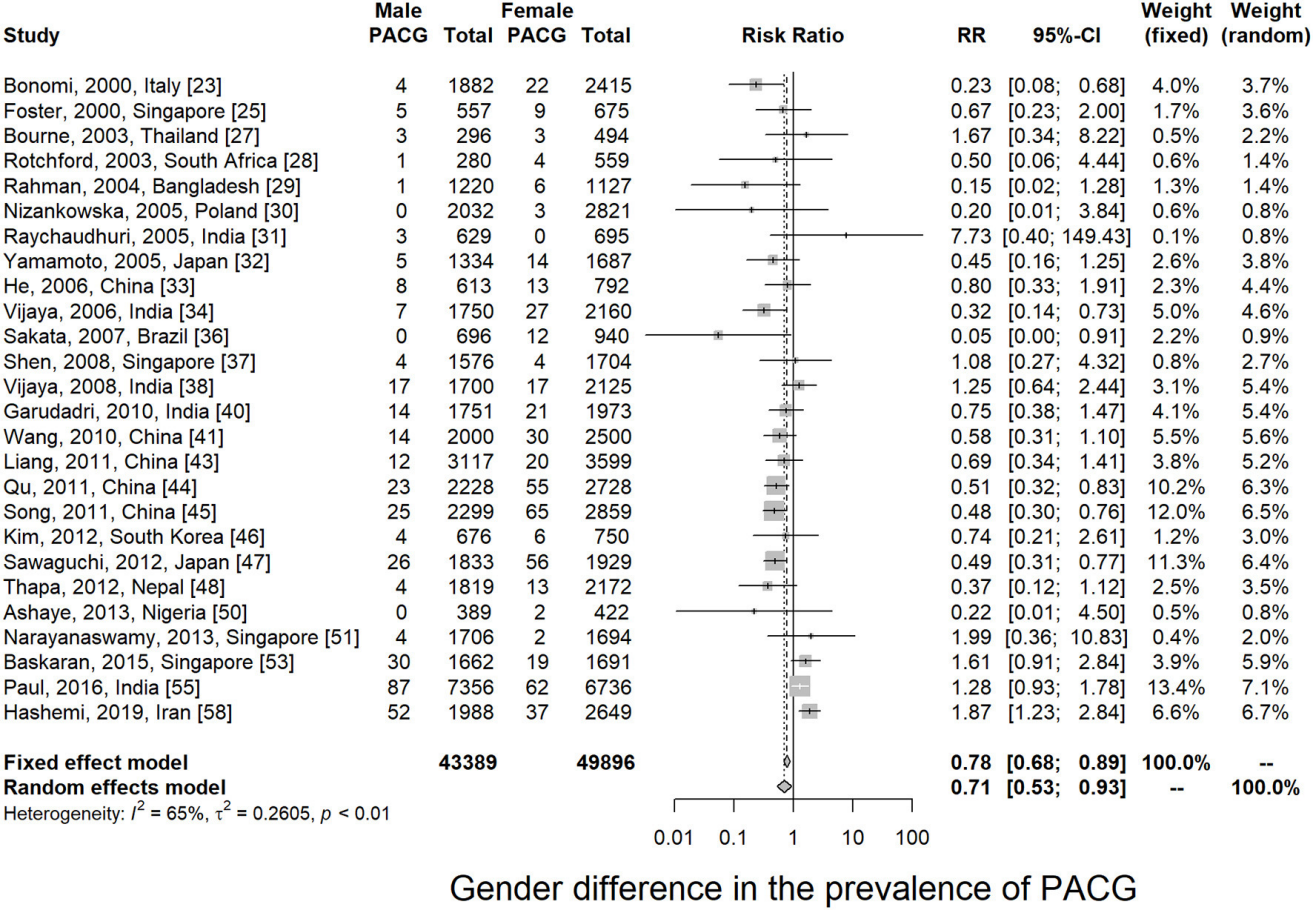
Sex comparison of primary angle-closure glaucoma. PACG, primary angle-closure glaucoma.

**Figure 4:**
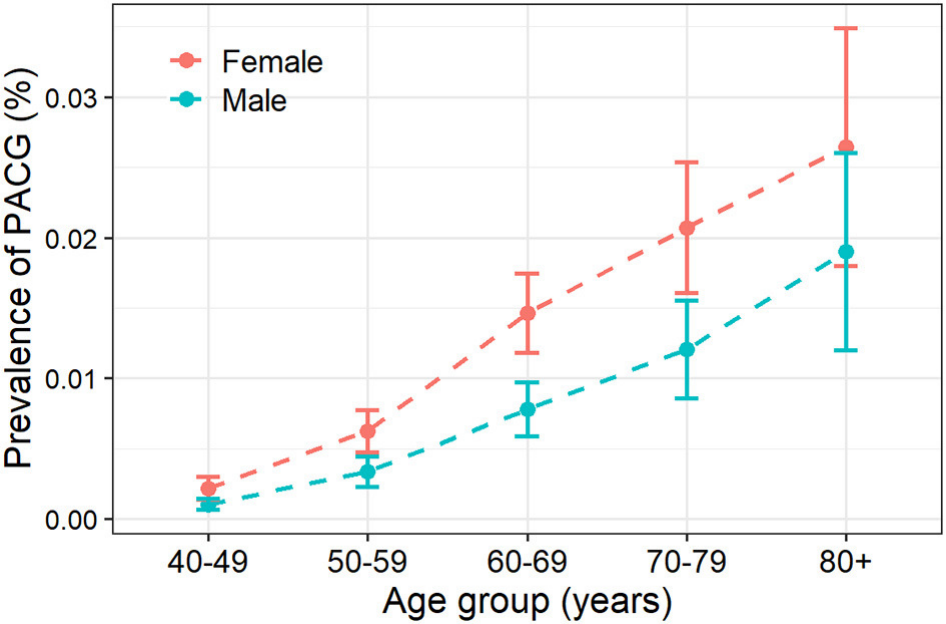
Prevalence of primary angle-closure glaucoma increased with aging. PACG, primary angle-closure glaucoma.

Twenty-six articles presented prevalence data by sex. Prevalence was higher for women in 69.2% of the studies (18 of 26). The male-to-female portions were ranged from 0.50 (Rotchford, 2003, South Africa) (28) to 1.09 (Paul, 2015, India) (55). Sex-specific prevalence of PACG is provided in Table 1; Supplementary Figure 5. This meta-analysis showed men are less likely to suffer from PACG than women with a relative risk of 0.71 (95% CI = 0.53–0.93, *p* < 0.01) in Figure 3. As summarized in Figure 4, the prevalence of PACG in the female sex is higher than the male sex in every age group. Subgroup differences test by random-effects model resulted in a significant difference between the prevalence of male and female groups (*Q* = 70.59, df = 25, *p* < 0.001).

Twenty-one studies reported age-specific prevalence of PACG; the detailed prevalence for each age group is listed in Table 1. Prevalence of PACG increased with aging steadily (Figure 4; Supplementary Figure 6). People older than 80 years old have highest prevalence (2.8%, 95% CI = 1.7–4.7%, *p* < 0.01). People aged 40–49 years have the lowest prevalence compared with other age groups (0.1%, 95% CI = 0.1–0.3%, *p* < 0.01). Subgroup differences tested using the random-effects model revealed a statistically significant difference among different age groups (*Q* = 64.71, df = 6, *p* < 0.001).

Most of the surveys we included in this study were conducted in Asia (28 of 37). A survey from Oceania and North America was lacking. Among all continents, Asia is found to have the highest prevalence of PACG (0.7%, 95% CI = 0.6–1.0%). South America has the same prevalence as Asia (0.7%, 95% CI = 0.4–1.3%). Europe has the lowest PACG prevalence compared with others (0.2%, 95% CI = 0.1–0.6%). Detailed prevalence of each continent is provided in Table 1; Supplementary Figure 7. Subgroup differences tested using the random-effects model revealed a statistically significant difference among different continents (*Q* = 12.84, df = 3, *p* = 0.005).

In this meta-analysis, 9 studies were conducted in urban, 15 in rural, and 17 were unknown or mixed. The prevalence of urban or rural population is listed in Table 1; Supplementary Figure 8. No statistical difference has been found between rural and urban populations using the random-effects model (*p* = 0.2387, *Q* = 2.87, df = 2).

### Risk Factors of Primary Angle-Closure Glaucoma

In this meta-analysis, we analyzed the prevalence of PACG according to various risk factors. Female sex (*Q* = 70.59, *p* < 0.001), Asian (*Q* = 12.84, *p* = 0.005), and aging (*Q* = 64.71, *p* < 0.001) are main risk factors of PACG.

### Number of People With Primary Angle-Closure Glaucoma Worldwide in 2020

The estimated number of aged populations (older than 40 years old) with PACG worldwide in 2020 and the next few decades are provided in Table 2. The populations with PACG are estimated based on our results and estimated world population number from the United Nations (20). The global population of PACG is 17.14 million (95% CI = 14.28–22.85) for population older than 40 years old in 2020, 20.73 million (95% CI = 17.27–27.63) in 2030, 23.73 million (95% CI = 19.78–31.64) in 2040, and 26.26 million (95% CI = 21.88–35.01) in 2050. Asia has the highest population of PACG among all continents in 2020 (12.30 million, 95% CI = 10.54–17.57) and also in the next few decades, accounts for more than 70% of the PACG population worldwide.

**Table 2:** Estimated global population of PACG.

**Continent**	**Estimated PACG cases (million, 95% CI)**			
	**2020**	**2030**	**2040**	**2050**
Africa	1.07 (0.53; 1.33)	1.49 (0.74; 1.86)	2.02 (1.01; 2.52)	2.71 (1.36; 3.39)
Asia	14.05 (10.54; 17.57)	15.02 (12.87; 21.45)	17.07 (14.63; 24.38)	18.47 (15.83; 26.39)
Europe	0.80 (0.40; 2.40)	0.85 (0.42; 2.54)	0.85 (0.42; 2.54)	0.83 (0.41; 2.48)
S. America	1.14 (0.65; 2.12)	2.06 (1.18; 3.84)	2.44 (1.40; 4.54)	2.73 (1.56; 5.06)
World	17.14 (14.28; 22.85)	20.73 (17.27; 27.63)	23.73 (19.78; 31.64)	26.26 (21.88; 35.01)

*PACG, primary angle-closure glaucoma; S. America, South America*.

## Discussion

This study provided the most updated worldwide prevalence of PACG for the last 20 years. Based on our results, the overall pooled PACG prevalence worldwide is 0.6% (95% CI = 0.5–0.8%). Asia has the highest PACG prevalence among all continents (0.7%, 95% CI = 0.6–1.0%). We estimated that the population of PACG is 17.14 million (95% CI = 14.28–22.85) for people older than 40 years old in 2020 globally, of which Asia accounts for over 70%. Our estimated PACG prevalence is similar to Tham et al.'s study, which reported the pooled PACG prevalence is 0.50% (95% CI = 0.11–1.36%) (6). PACG is still a worldwide public health burden that requires improvement in diagnostic and therapeutic approaches, particularly in Asia. The risk factors for PACG, including age, sex, and ethnicity, were discussed in detail as follows.

### Age

Age is known to be the major risk factor for all types of glaucoma, as the prevalence increase with age (7, 60, 61). This is confirmed in this meta-analysis. Aging per decade is consistently associated with higher intraocular pressure, thinner central corneal thickness, and higher mean ocular perfusion pressure (62). For PACG pathogenesis, multiple mechanisms contributed to angle closure, including pupillary block and plateau iris, resulting in increased intraocular pressure and neurodegeneration (63). Anatomical changes could explain the increase of morbidity, and narrow anterior chamber depth (ACD) increases the risk of PACG. ACD and anterior chamber area both significantly decreased with age (−0.0119 mm/year, −0.0845 mm^2^/year, *p* < 0.0001), which was caused by increment of iris cross-sectional area, iris curvature, and lens vault (64). Besides the lens becomes more compact and thicker with increasing age, proportionately large lens contributed to pupillary block and angle-crowding (65). Moreover, morphological studies have indicated that the outflow ability decreased with age and resulted in increased intraocular pressure, which was caused by the accumulation of extracellular materials in trabecular meshwork (66).

### Sex

In this study, sex is found to be a significant risk factor for PACG; females are more likely to have PACG than males at all age groups (Figures 3, 4). Various studies had associated shallow anterior chamber and narrow chamber angle with female sex (67–69). Moreover, females were shown to have greater ACD shallowing with aging than males (70). The mean ACD values were significantly different from men [2.59 mm (2.56; 2.62)] to women [2.42 mm (2.39; 2.44)] in elderly Chinese (older than 50 years old) (71). Such anatomical differences could contribute to the sex difference in PACG. Other factors such as endocrinologic difference and menopausal status might also be involved in sex differences for the prevalence of PACG (72).

### Ethnicity and Continent

In this meta-analysis, most of the included studies (28 of 37) were conducted in Asian countries. Although the majority of the ethnicity from Asian countries are from Asia, people from other countries such as the Europeans were of mixed ethnicity. Because most of the studies lack detailed prevalence data for each ethnicity, it is not possible to perform a meta-analysis for ethnicity based on such limited information. Hence, continent differences were analyzed instead.

As we mentioned earlier, the majority of the population from Asian countries are Asians. The results from the continent of Asia may represent the prevalence of PACG for Asians (0.7%, 95% CI = 0.6–1.0%, *p* < 0.01). It is previously reported that Asians have a higher prevalence of PACG (73, 74), consistent with findings from this meta-analysis (Table 1; Supplementary Figure 7). Chan et al. reported that the PACG prevalence in Asia was 0.73% (95% CI = 0.18–1.96%) in 2013, which is similar to our results (75). They also estimated that the population with PACG would be 13.43 million (95% CI = 4.01–31.79) in 2020 and 17.51 million (95% CI = 5.21–41.37) in 2040. Our estimated PACG prevalence of Asia is slightly lower than Tham et al. (1.09%, 95% CI = 0.43–2.32%) (6), which might be due to the newly included seven studies (51–53, 55–58) conducted after the year 2012, which accounted for more than half of the Asian participants (64,380 of 110,833) in this meta-analysis. Our study provides a more up-to-date PACG prevalence. Anatomical differences might be contributed to the high prevalence of PACG in Asians. A prospective study from the United States found that Chinese–American people had a significantly thick iris at 750 and 2,000 μm from the scleral spurs (76). Another reported that Chinese and Hispanic subjects had the highest mean value of iris thickness at 750 μm from the scleral spurs, lowest anterior chamber area, anterior chamber volume, and anterior chamber width compared with Whites and Africans (77). The prevalence of PACG also varies in different Asian regions. South-central Asia was considered to have the highest overall glaucoma and primary open-angle glaucoma burden than other regions, whereas East Asia has a higher PACG prevalence (75).

### Habitation Area

Besides sex, age, and continents, habitation area (urban or rural) was also analyzed in this study (Supplementary Figure 8). No statistical difference was found in the prevalence of PACG between rural and urban populations. However, this part of the analysis represents substantial bias for the following reasons: (1) The information habitation area is usually vaguely described in the majority of the studies; (2) There are only a few studies that have included both urban and rural populations in the study, and therefore, the comparison between urban and rural across studies represent ethnicity and country bias. The only study that has reported the prevalence of PACG for both habitation areas is Paul et al.'s study of the Indian population in 2016 (55). They showed that the prevalence of PACG is slightly higher in the rural (1.15%) than urban area (0.97%). However, because our meta-analysis represents bias for the reasons mentioned earlier, more evidence is needed to reveal the role of the habitation area in the risk of PACG in future studies.

### Bias and Heterogeneity

The risk of bias in this meta-analysis was from the following three major aspects: the selection of participants, response rate, and diagnostic criteria for the outcome measurement (Supplementary Table 3). The overall risk of bias for this study is low because low-quality studies were excluded, as mentioned in the method. In this meta-analysis, the overall heterogeneity is high (*I*^2^ = 94.4%). Commonly, a meta-analysis for prevalence studies yields very high heterogeneities, usually more than 90% of the *I*^2^ value (22, 78–81). The impact of study quality on pooled prevalence was assessed by excluding low-quality studies and by conducting a meta-regression, comparing studies at low risk of bias with those at moderate-to-high risk. Meta-regression demonstrated little evidence of risk of bias, giving a consistent level of prevalence. It is noted that in this meta-analysis, the heterogeneity dropped dramatically in people with the age of 80+ years (*I*^2^ = 25.3%) and among studies from Africa (*I*^2^ = 46.3%, Table 1), indicating that the risk factors of age and geographical location are possibly the main sources of the heterogeneity.

## Limitations

The major limitation of this study is that the number of studies conducted in the last 20 years varies a lot across continents, and therefore, the overall prevalence for some continents represents selection bias. There is only one study for South America (36) and three studies for Europe (23, 30, 59). The data from North America and Oceania lack in this meta-analysis.

## Conclusion

In this meta-analysis, we reviewed 37 studies of 144,354 subjects for the prevalence of PACG in the last 20 years. Up to date, PACG is still a worldwide vision-threatening disease with high prevalence (0.6%, 95% CI = 0.5–0.8%), which is affecting about 17.14 million aged people in the world, especially in Asia (12.30 million). Asian, female sex, and aging are considered to be risk factors of PACG. Early screening in people with high risks is needed in early intervention of PACG, particularly in Asian countries.

## Data Availability Statement

The original contributions presented in the study are included in the article/Supplementary Material, further inquiries can be directed to the corresponding author/s.

## Author Contributions

NZ, JW, and BJ: research design. NZ, JW, and BC: data acquisition, research execution, and data analysis. NZ, JW, BC, YL, and BJ: manuscript preparation. All authors: contributed to the article and approved the submitted version.

## Conflict of Interest

The authors declare that the research was conducted in the absence of any commercial or financial relationships that could be construed as a potential conflict of interest.
