# Supplementary material for: Prevalence of Primary Angle Closure Glaucoma in the Last 20 Years: A Meta-Analysis and Systematic Review
Source: Front Med (Lausanne). 2021 Jan 18;7:624179. doi: 10.3389/fmed.2020.624179 (PMC7847989; doi:10.3389/fmed.2020.624179)
Supplement: Supplementary file 1 [file Data_Sheet_1.docx]

**Supplementary Materials**

Supplementary Table 1. Database specific search strategy.

| Database | Search strategy |
| --- | --- |
| PubMed | 1. ("glaucoma, angle-closure" [Mesh]) AND (("prevalence" [Mesh]) OR ("population" [Mesh]) OR ("epidemiology" [Mesh]) 2. (("primary angle closure glaucoma" [Title/Abstract]) OR ("primary angle-closure glaucoma" [Title/Abstract]) OR ("PACG" [Title/Abstract])) AND (("prevalence" [Title/Abstract]) OR ("Survey" [Title/Abstract]) OR ("population" [Title/Abstract]) OR ("epidemiology" [Title/Abstract])) 3. "2000"[Date - Publication]: "2020"[Date - Publication]   (1 OR 2) AND 3 |
| Embase | ('primary angle closure glaucoma': ab,ti OR 'primary angle-closure glaucoma':ab,ti OR pacg: ab,ti) AND (prevalence: ab,ti OR survey: ab,ti OR population: ab,ti OR epidemiology: ab,ti) AND [2000-2020]/py |
| Web of Science | 1. TI= (((primary angle closure glaucoma) OR (primary angle-closure glaucoma) OR (PACG)) AND ((prevalence) OR (survey) OR (population) OR (epidemiology)))   **Timespan:** 2000-2020. **Databases:**  WOS, BCI, KJD, MEDLINE, RSCI, SCIELO.  Search language=Auto   1. AB= (((primary angle closure glaucoma) OR (primary angle-closure glaucoma) OR (PACG)) AND ((prevalence) OR (survey) OR (population) OR (epidemiology)))   **Timespan:** 2000-2020. **Databases:**  WOS, BCI, KJD, MEDLINE, RSCI, SCIELO.  Search language=Auto   1 OR 2 |

Supplementary Table 2. Characteristics of included studies.

Supplementary Table 2. Characteristics of included studies. (continued)

Supplementary Table 3. Guidelines for assessing risk of bias – based on study participation and outcome measurement domains of the QUIPS tool [1].

| **Potential bias** | **Items to be considered for assessment of potential bias** | **Items for risk ratings** |
| --- | --- | --- |
| **Study participation**  Does the study sample represent the population of interest? | 1. Description of the baseline study sample. Is this study population based and randomized sampling? 2. Description of the source population or population of interest 3. Adequate description of the source population of interest, the sampling frame and place of recruitment 4. Is there adequate participation in the study? 5. Response rate 6. Participants selection biased by potential risk factors for PACG, such as age, gender and ethnicity. | **High risk of bias:**  The relationship between the PF and outcome is very likely to be different for  participants and eligible nonparticipants  Age inclusion of only 60+  **Moderate risk of bias**  The relationship between the PF and outcome may be different for participants  and eligible nonparticipants  Only a certain minority group was included  Age inclusion of only 50+  **Low risk of bias**  The relationship between the PF and outcome is unlikely to be different for participants and eligible nonparticipants  Age inclusion of 40+ |
| **Outcome measurement**  Is the outcome of interest  measured in a similar  way for all participants? | 1. Whether the ISGEO2002 diagnosis criteria were used for definition of PACG 2. Whether a clear definition of PACG is provided if ISGEO2002 criteria is not used in the study 3. Whether the method of PACG definition used is adequately valid and reliable 4. Whether the method is the same for all study participants 5. Is there possible involvement of other types of angle closure glaucoma (ACG) in the reported prevalence? | **High risk of bias:**  The measurement of the PF is very likely  to be different for different levels of the outcome of interest  **Moderate risk of bias**  The measurement of the PF may be different for different levels of the outcome of interest  **Low risk of bias**  The measurement of the PF is unlikely  to be different for different levels of the outcome of interest |

Supplementary Table 4. Risk of selection and outcome measurement bias and justification for rating using the QUIPS tool.

| **Study** | **Risk of study participation bias** | **Risk of outcome measurement bias** |
| --- | --- | --- |
| **Bonomi, 2000, Italy [23]** | **Moderate**  Randomized, population-based sampling for subjects with age of 40+. Response rate was relatively low at 74% and therefore considered moderate risk for selection bias. | **Moderate**  Prevalence of PACG was reported. Diagnosis criteria of ISGEO2002 was not used and therefore considered of moderate risk for outcome measurement bias. The diagnosis of PACG was made on the basis of the concomitant presence of at least two of the following criteria: intraocular pressure ≥ 22 mmHg, glaucomatous optic disc abnormalities, glaucomatous visual field defects. |
| **Buhrmann, 2000, Tanzania [24]** | **Low**  Randomized, population-based sampling for subjects with age of 40+. Response rate was 90% and therefore considered low risk for selection bias. | **Moderate**  Prevalence of PACG was reported. Diagnosis criteria of ISGEO2002 was not used and therefore considered of moderate risk for outcome measurement bias.  Primary ACG was diagnosed in a person if one eye had primary occludable angle and also had one or more of the following criteria in that eye: IOP higher than 24 mm Hg, structural optic disc abnormality, definite, reliable visual field damage, or a history compatible with an episode of acute angle closure or the development of an episode at examination |
| **Foster, 2000, Singapore [25]** | **Moderate**  Disproportionate, stratified, clustered, random-sampling for subjects with age of 40+. Response rate was relatively low at 71.8% and therefore considered moderate risk for selection bias. | **Moderate**  Prevalence of PACG was reported. Diagnosis criteria of ISGEO2002 was not used and therefore considered of moderate risk for outcome measurement bias.  Full ocular examination including visual acuity, visual field, near-refractive correction, screening-mode frequency doubling technology test, anterior segment, IOP, Gonioscopy and fundus were performed. Very detailed diagnostic criteria were provided in the study. |
| **Rotchford, 2002, South Africa [26]** | **Low**  Two-stage, non-stratified, cluster-based, random-sampling for subjects with age of 40+. Response rate was 90% and therefore considered low risk for selection bias. | **Low**  Prevalence of PACG was reported. ISGEO2002 criteria was used for diagnose. Therefore, considered as low risk for outcome measurement bias |
| **Bourne, 2003, Thailand [27]** | **Moderate**  Randomized, population-based sampling for subjects with age of 50+. The sampling process requires the individuals in a household had to own the home  and individuals selected had to have no intention of moving from the area within 3 years. Therefore, this study was considered moderate risk for selection bias. Response rate was high at 88.5%. | **Low**  Prevalence of PACG was reported. ISGEO2002 criteria was used for diagnose. Therefore, considered as low risk for outcome measurement bias |
| **Rotchford, 2003, South Africa [28]** | **Moderate**  Two-stage non-stratified, cluster-based random sampling for subjects with age of 40+. Response rate was relatively low at 74% and therefore considered moderate risk for selection bias. | **Low**  Prevalence of PACG was reported. ISGEO2002 criteria was used for diagnose. Therefore, considered as low risk for outcome measurement bias |
| **Rahman, 2004, Bangladesh [29]** | **Moderate**  Multistage, stratified, clustered sampling for subjects with age of 35+. Response rate was low of 66% and therefore considered moderate risk for selection bias. | **Low**  Prevalence of PACG was reported. ISGEO2002 criteria was used for diagnose. Therefore, considered as low risk for outcome measurement bias |
| **Nizankowska, 2005, Poland [30]** | **Low**  Proportional simple random sampling selection with stratification for subjects with age of 40+. Response rate was 81% and therefore considered low risk for selection bias. | **Moderate**  Prevalence of PACG was reported. Diagnosis criteria of ISGEO2002 was not used and therefore considered of moderate risk for outcome measurement bias.  Glaucoma was diagnosed by the presence of any two of the following: characteristic morphological changes in the optic disc, glaucomatous visual field abnormalities, and intraocular pressure greater than 21 mmHg. |
| **Raychaudhuri, 2005, India [31]** | **Moderate**  Randomized, population-based sampling for subjects with age of 50+ and therefore was considered moderate risk for selection bias. Response rate was high at 83.1%. | **Low**  Prevalence of PACG was reported. ISGEO2002 criteria was used for diagnose. Therefore, considered as low risk for outcome measurement bias |
| **Yamamoto, 2005, Japan [32]** | **Low**  Randomized, population-based sampling for subjects with age of 40+. Response rate was 78.1% and therefore considered low risk for selection bias. | **Low**  Prevalence of PACG was reported. ISGEO2002 criteria was used for diagnose. Therefore, considered as low risk for outcome measurement bias |
| **He, 2006, China [33]** | **Moderate**  Random clustering sampling for subjects with age of 50+ and therefore was considered moderate risk for selection bias. Response rate was high at 75.3%. | **Low**  Prevalence of PACG was reported. ISGEO2002 criteria was used for diagnose. Therefore, considered as low risk for outcome measurement bias |
| **Vijaya, 2006, India [34]** | **Low**  Randomized, population-based sampling for subjects with age of 40+. Response rate was 81.75% and therefore considered low risk for selection bias. | **Low**  Prevalence of PACG was reported. ISGEO2002 criteria was used for diagnose. Therefore, considered as low risk for outcome measurement bias |
| **Casson, 2007, Myanmar [35]** | **Low**  Randomized, population-based sampling for subjects with age of 40+. Response rate was 80.5% and therefore considered low risk for selection bias. | **Low**  Prevalence of PACG was reported. ISGEO2002 criteria was used for diagnose. Therefore, considered as low risk for outcome measurement bias |
| **Sakata, 2007, Brazil [36]** | **Low**  Randomized, population-based sampling for subjects with age of 40+. Response rate was 76.5% and therefore considered low risk for selection bias. | **Low**  Prevalence of PACG was reported. ISGEO2002 criteria was used for diagnose. Therefore, considered as low risk for outcome measurement bias |
| **Shen, 2008, Singapore [37]** | **Low**  Age-stratified, random sampling for subjects with age of 40+. Response rate was 78.7% and therefore considered low risk for selection bias. | **Low**  Prevalence of PACG was reported. ISGEO2002 criteria was used for diagnose. Therefore, considered as low risk for outcome measurement bias |
| **Vijaya, 2008, India [38]** | **Low**  Randomized, population-based sampling for subjects with age of 40+. Response rate was 80.2% and therefore considered low risk for selection bias. | **Low**  Prevalence of PACG was reported. ISGEO2002 criteria was used for diagnose. Therefore, considered as low risk for outcome measurement bias |
| **Casson, 2009, Sri Lanka [39]** | **Low**  Randomized, population-based sampling for subjects with age of 40+. Response rate was 79.9% and therefore considered low risk for selection bias. | **Low**  Prevalence of PACG was reported. ISGEO2002 criteria was used for diagnose. Therefore, considered as low risk for outcome measurement bias |
| **Garudadri, 2010, India [40]** | **Low**  Stratified, random, cluster, and systematic sampling for subjects with age of 40+. Response rate was 88% and therefore considered low risk for selection bias. | **Low**  Prevalence of PACG was reported. ISGEO2002 criteria was used for diagnose. Therefore, considered as low risk for outcome measurement bias |
| **Wang, 2010, China [41]** | **Low**  Randomized, population-based sampling for subjects with age of 40+. Response rate was 83.4% and therefore considered low risk for selection bias. | **Low**  Prevalence of PACG was reported. ISGEO2002 criteria was used for diagnose. Therefore, considered as low risk for outcome measurement bias. |
| **AI-Mansouri, 2011, Qatar [42]** | **Low**  Random selected clusters for subjects with age of 40+. Response rate was 97.3% and therefore considered low risk for selection bias. | **Moderate**  Prevalence of PACG was reported. Diagnosis criteria of ISGEO2002 was not used and therefore considered of moderate risk for outcome measurement bias.  Glaucoma was defined as either eye had (1) evidence of glaucomatous cup or surrounding retina showed signs of glaucoma and (2) intraocular pressure of ≥22 mmHg measured with applanation tonometry |
| **Liang, 2011, China [43]** | **Low**  Randomized, population-based sampling for subjects with age of 30+. Response rate was 98.3% and therefore considered low risk for selection bias. | **Low**  Prevalence of PACG was reported. ISGEO2002 criteria was used for diagnose. Therefore, considered as low risk for outcome measurement bias |
| **Qu, 2011, China [44]** | **Low**  Randomized, stratified, cluster sampling for subjects with age of 40+. Response rate was 86.01% and therefore considered low risk for selection bias. | **Low**  Prevalence of PACG was reported. ISGEO2002 criteria was used for diagnose. Therefore, considered as low risk for outcome measurement bias |
| **Song, 2011, China [45]** | **Low**  Randomized, population-based sampling for subjects with age of 40+. Response rate was 87.4% and therefore considered low risk for selection bias. | **Low**  Prevalence of PACG was reported. ISGEO2002 criteria was used for diagnose. Therefore, considered as low risk for outcome measurement bias |
| **Kim, 2012, South Korea [46]** | **Low**  Randomized, population-based sampling for subjects with age of 40+. Response rate was 79.5% and therefore considered low risk for selection bias. | **Low**  Prevalence of PACG was reported. ISGEO2002 criteria was used for diagnose. Therefore, considered as low risk for outcome measurement bias |
| **Sawaguchi, 2012, Japan [47]** | **Low**  Randomized, population-based sampling for subjects with age of 40+. Response rate was 81.2% and therefore considered low risk for selection bias. | **Low**  Prevalence of PACG was reported. ISGEO2002 criteria was used for diagnose. Therefore, considered as low risk for outcome measurement bias |
| **Thapa, 2012, Nepal [48]** | **Low**  Randomized, cluster sampling for subjects with age of 40+. Response rate was 83.4% and therefore considered low risk for selection bias. | **Low**  Prevalence of PACG was reported. ISGEO2002 criteria was used for diagnose. Therefore, considered as low risk for outcome measurement bias |
| **Zhong, 2012, China [49]** | **Moderate**  Randomized, population-based sampling for subjects with age of 50+ and therefore was considered moderate risk for selection bias. Response rate was high at 77.8%. | **Low**  Prevalence of PACG was reported. ISGEO2002 criteria was used for diagnose. Therefore, considered as low risk for outcome measurement bias |
| **Ashaye, 2013, Nigeria [50]** | **Low**  Randomized, population-based sampling for subjects with age of 40+. Response rate was 90% and therefore considered low risk for selection bias. | **Low**  Prevalence of PACG was reported. ISGEO2002 criteria was used for diagnose. Therefore, considered as low risk for outcome measurement bias |
| **Narayanaswamy, 2013, Singapore [51]** | **Low**  Age-stratified random sampling for subjects with age of 40+. Response rate was 75.6% and therefore considered low risk for selection bias. | **Low**  Prevalence of PACG was reported. ISGEO2002 criteria was used for diagnose. Therefore, considered as low risk for outcome measurement bias |
| **Pakravan, 2013, Iran [52]** | **Low**  Randomized, cluster sampling for subjects with age of 40+. Response rate was 90.4% and therefore considered low risk for selection bias. | **Low**  Prevalence of PACG was reported. ISGEO2002 criteria was used for diagnose. Therefore, considered as low risk for outcome measurement bias |
| **Baskaran, 2015, Singapore [53]** | **Moderate**  Age-stratified random sampling for subjects with age of 40+. Response rate was 72.8% and therefore considered moderate risk for selection bias. | **Low**  Prevalence of PACG was reported. ISGEO2002 criteria was used for diagnose. Therefore, considered as low risk for outcome measurement bias |
| **Kyari, 2015, Nigeria [54]** | **Low**  Multistage stratified cluster random sampling for subjects with age of 40+. Response rate was 90.4% and therefore considered low risk for selection bias. | **Low**  Prevalence of PACG was reported. ISGEO2002 criteria was used for diagnose. Therefore considered as low risk for outcome measurement bias |
| **Paul, 2016, India [55]** | **Low**  Multistage random cluster sampling for subjects with age of 40+. Response rate was 98% and therefore considered low risk for selection bias. | **Low**  Prevalence of PACG was reported. ISGEO2002 criteria was used for diagnose. Therefore, considered as low risk for outcome measurement bias |
| **Chassid, 2018, Israel [56]** | **Low**  Randomized, population-based sampling for subjects with age of 40+. Response rate was 82% and therefore considered low risk for selection bias. | **Moderate**  Prevalence of PACG was reported. Diagnosis criteria of ISGEO2002 was not used and therefore considered of moderate risk for outcome measurement bias.  Diagnosis criteria for PACG was not clearly described in study. Only diagnosis criteria for POAG was provided as following:  Individuals were classified with POAG if they met any of the following diagnostic criteria: cup-to-disk ratio (CDR) >0.5 with asymmetry between the eyes and damage to the field of vision; intraocular pressure (IOP) >23 mm Hg; damage to the field of vision without CDR asymmetry, but with IOP >23 mm Hg; CDR asymmetry >0.5 with damage to the field of vision, independent of IOP; CDR asymmetry >0.2 and damage to the field of vision, with or without increased IOP; damage to the field of vision as defined by the following characteristics of glaucomatous field defects: asymmetry across the horizontal midline (in early/moderate cases), location in the mid-periphery (in early/moderate cases), clustering in neighboring test points, and reproducibility on at least two occasions. |
| **Addepalli, 2019, India [57]** | **Low**  Randomized, population-based sampling for subjects with age of 40+. Response rate was 86% and therefore considered low risk for selection bias. | **Low**  Prevalence of PACG was reported. ISGEO2002 criteria was used for diagnose. Therefore, considered as low risk for outcome measurement bias |
| **Hashemi, 2019, Iran [58]** | **Low**  Randomized, multistage cluster sampling for subjects with age of 40+. Response rate was 82.2% and therefore considered low risk for selection bias. | **Low**  Prevalence of PACG was reported. ISGEO2002 criteria was used for diagnose. Therefore, considered as low risk for outcome measurement bias |
| **McCann, 2020, Northern Ireland [59]** | **Moderate**  Randomized, population-based sampling for subjects with age of 40+. Response rate was not reported and therefore considered moderate risk for selection bias. | **Low**  Prevalence of PACG was reported. ISGEO2002 criteria was used for diagnose. Therefore, considered as low risk for outcome measurement bias |

1. Hayden, J.A., et al., *Assessing bias in studies of prognostic factors.* Ann Intern Med, 2013. **158**(4): p. 280-6.


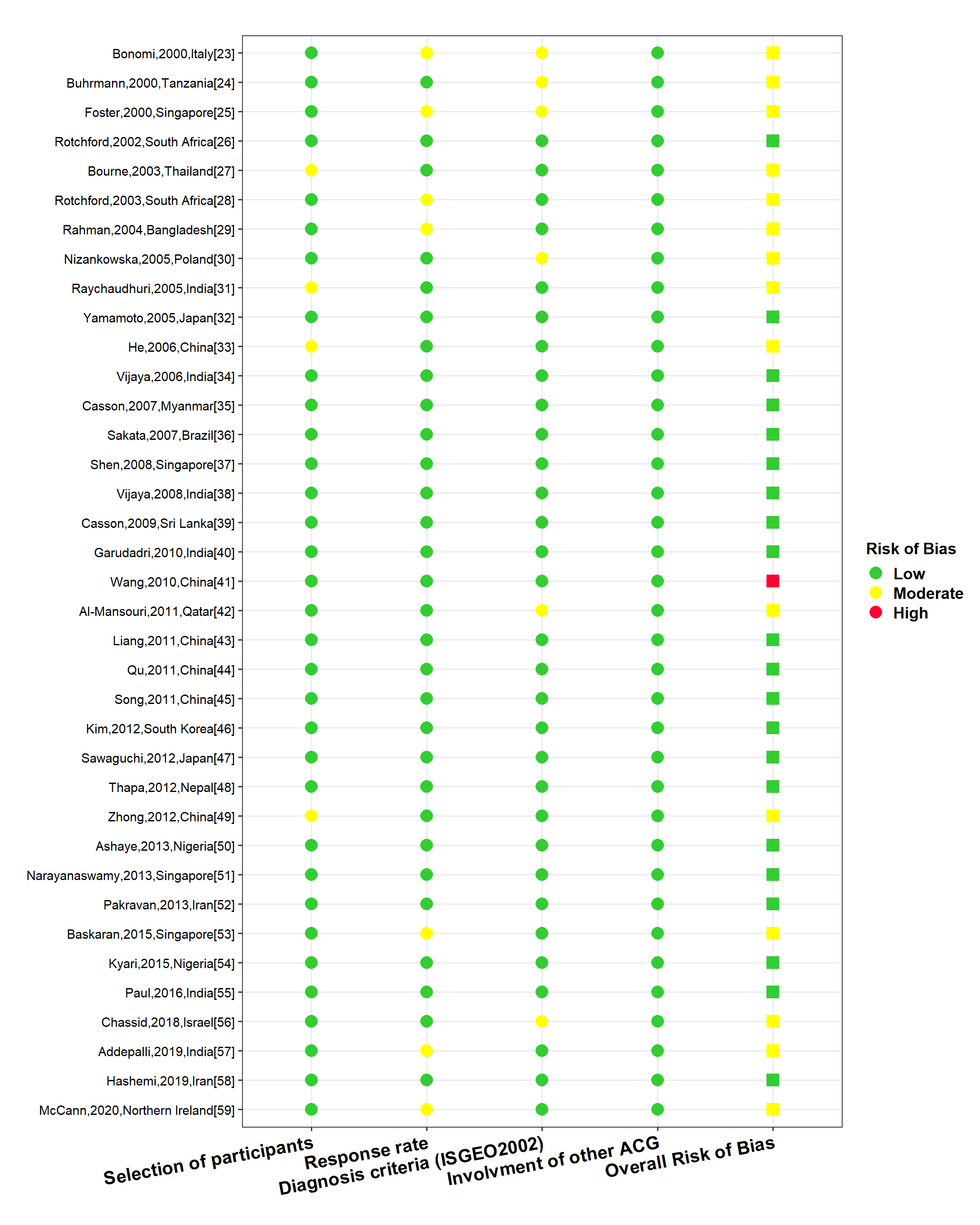


Supplementary Figure 1. Traffic lights for risk of bias.


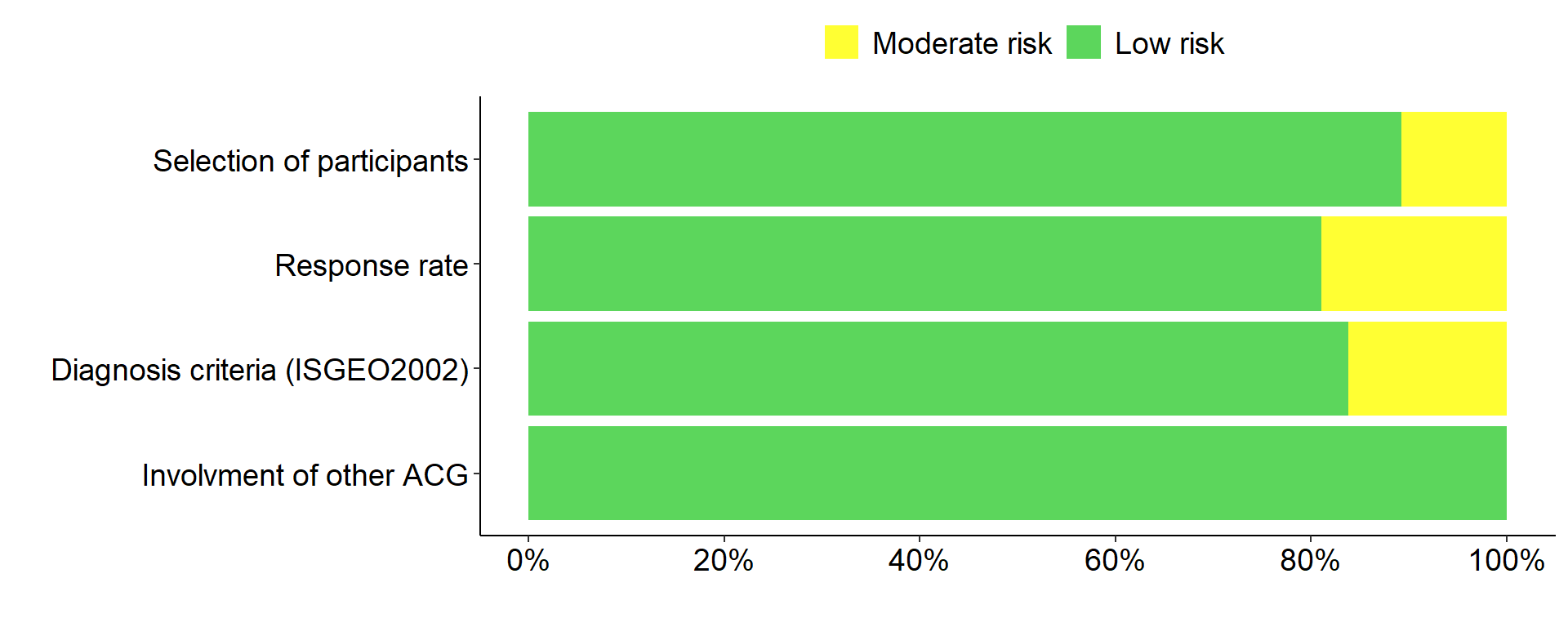


Supplementary Figure 2. Summary of risk of bias.


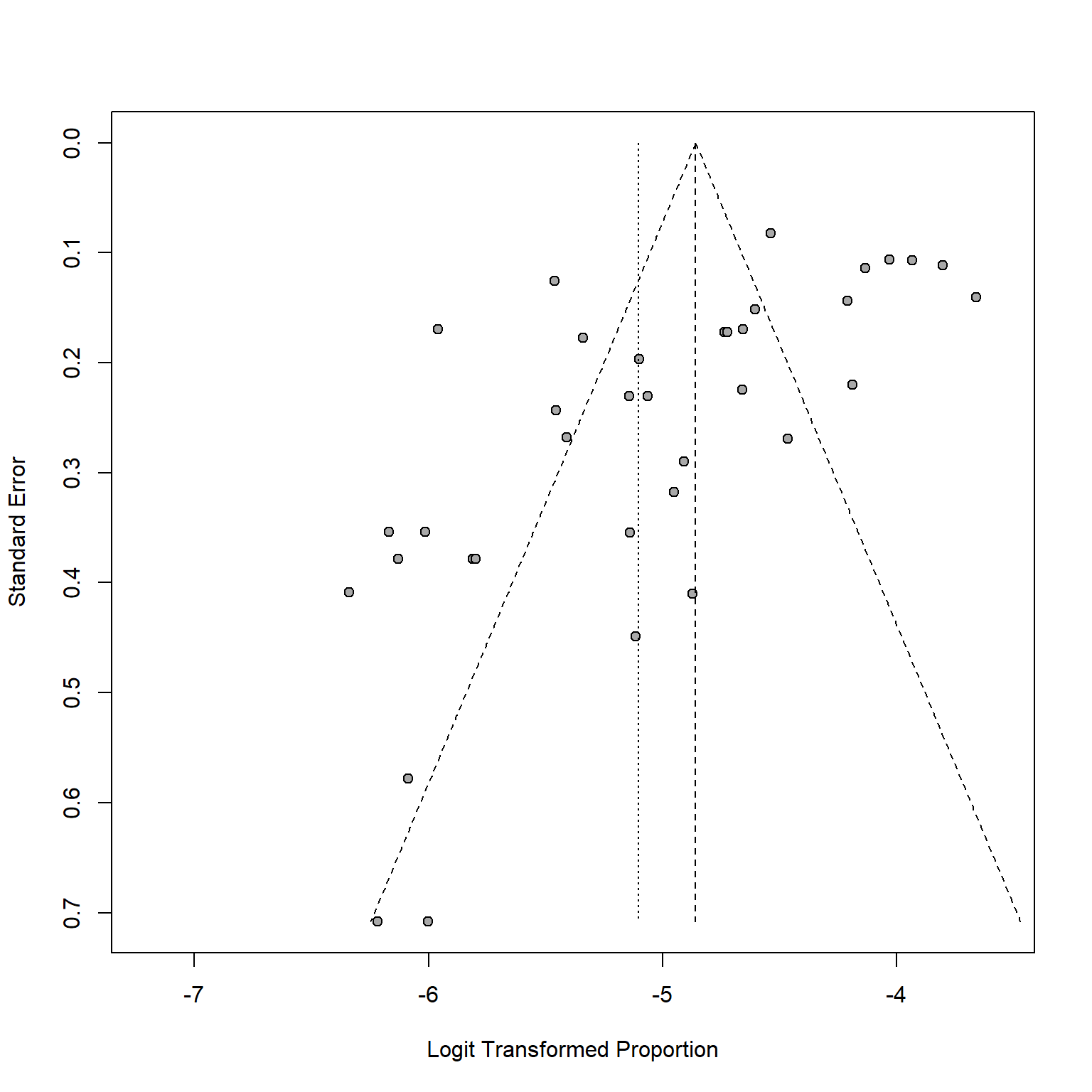
Supplementary Figure 3. Funnel plots for all studies.


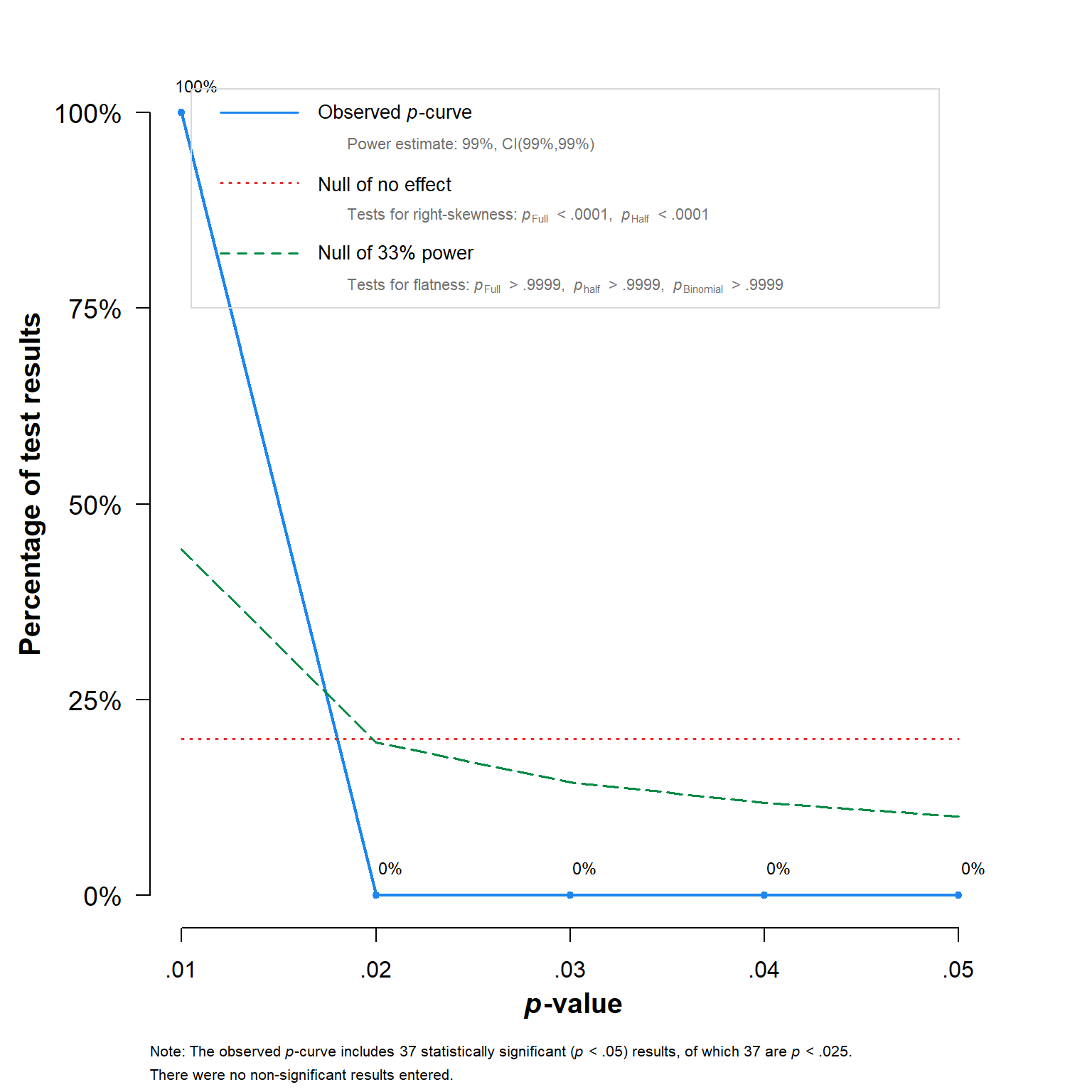
Supplementary Figure 4. P-value analysis for all studies.


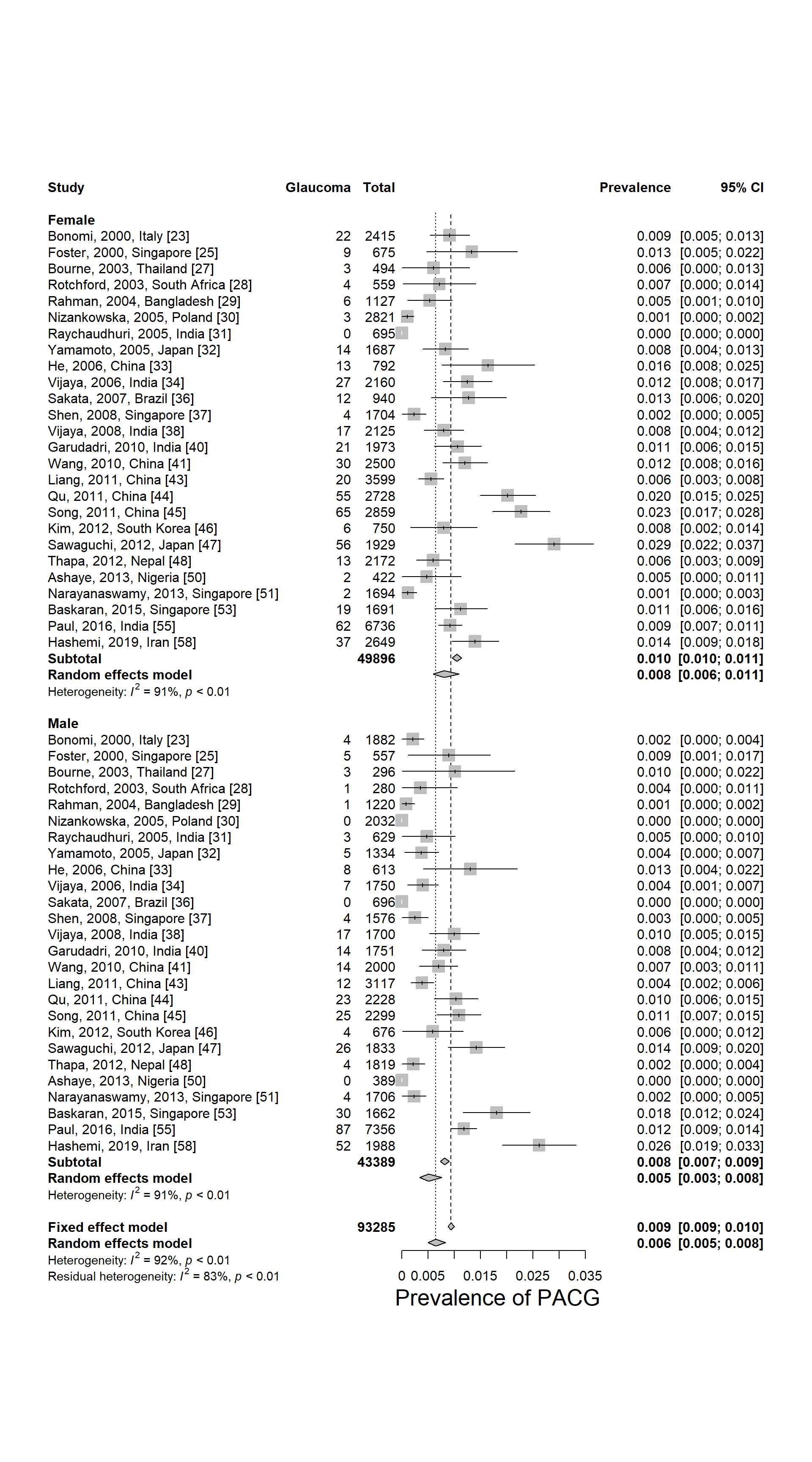


Supplementary Figure 5. PACG prevalence by gender groups.


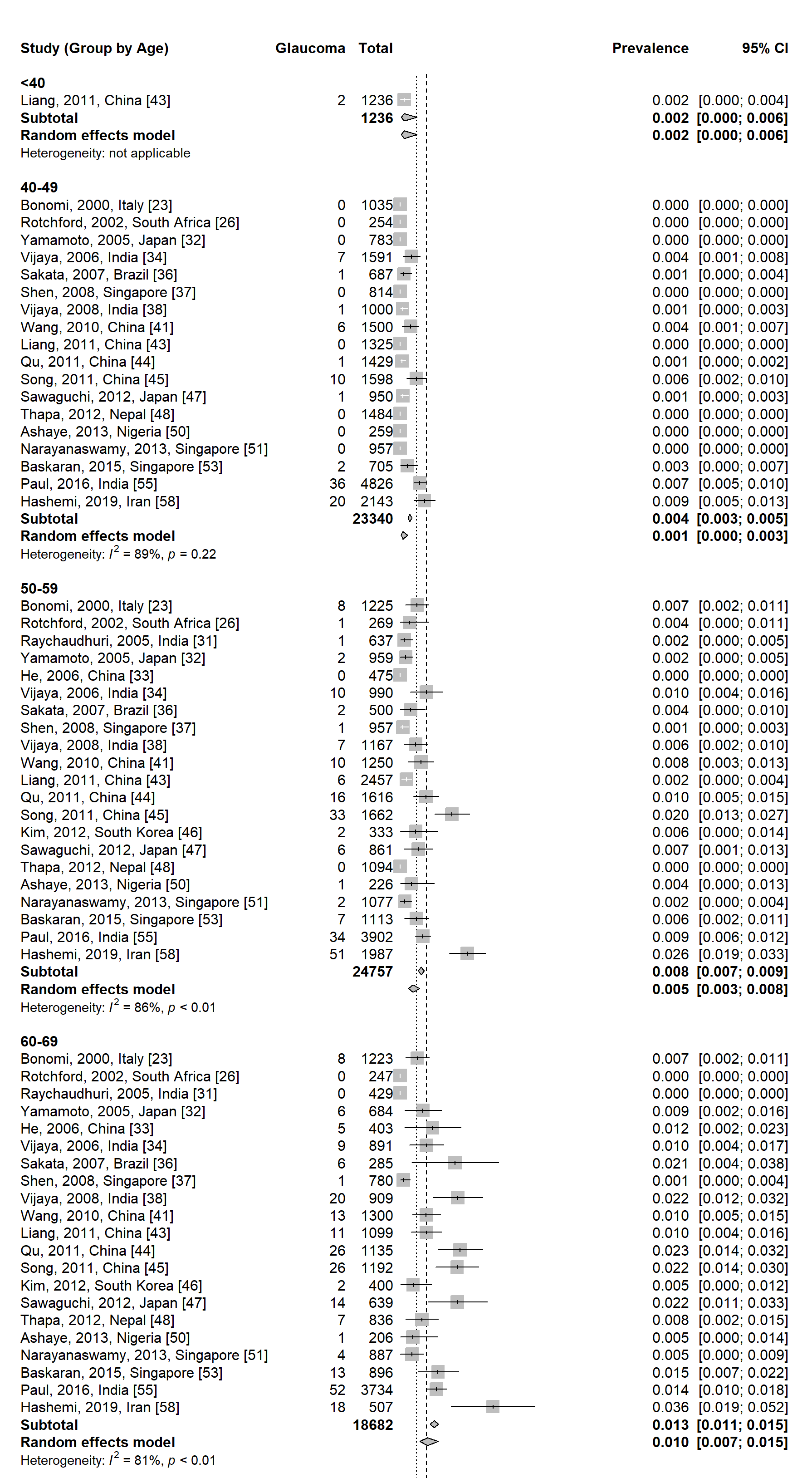


Supplementary Figure 6. PACG prevalence by age groups.


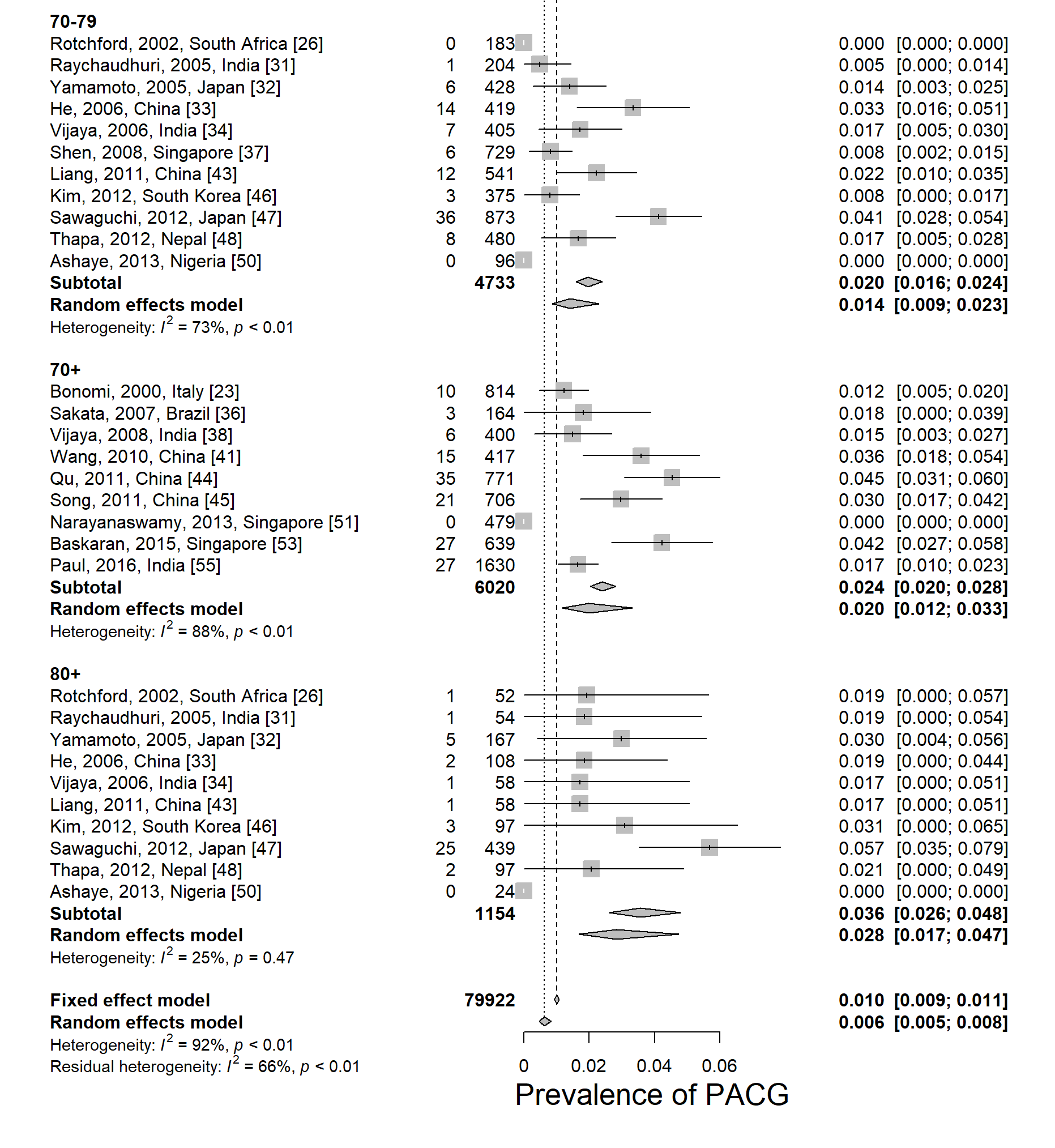


Supplementary Figure 6. (continued) PACG prevalence by age groups.


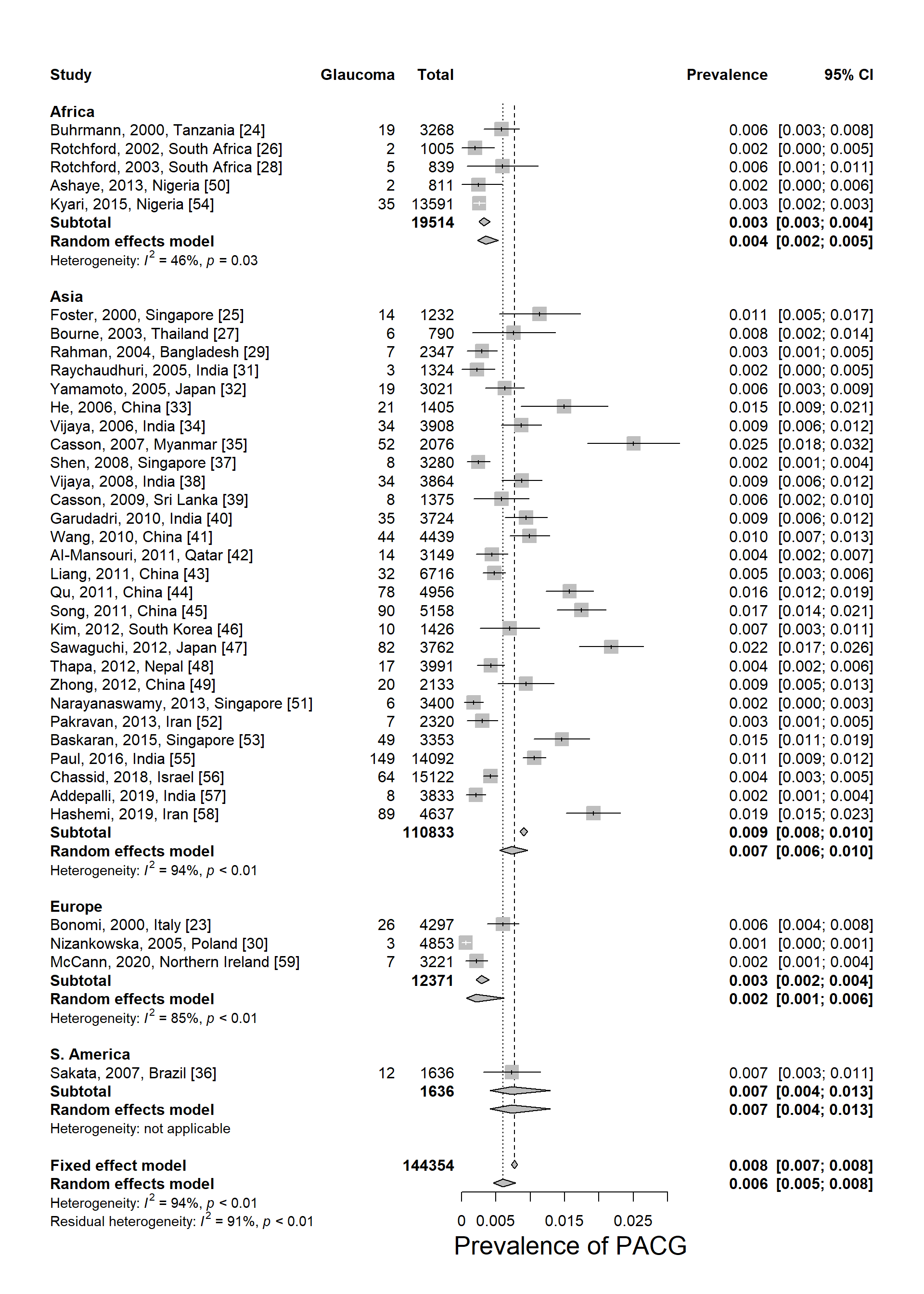
Supplementary Figure 7. PACG prevalence by continents.


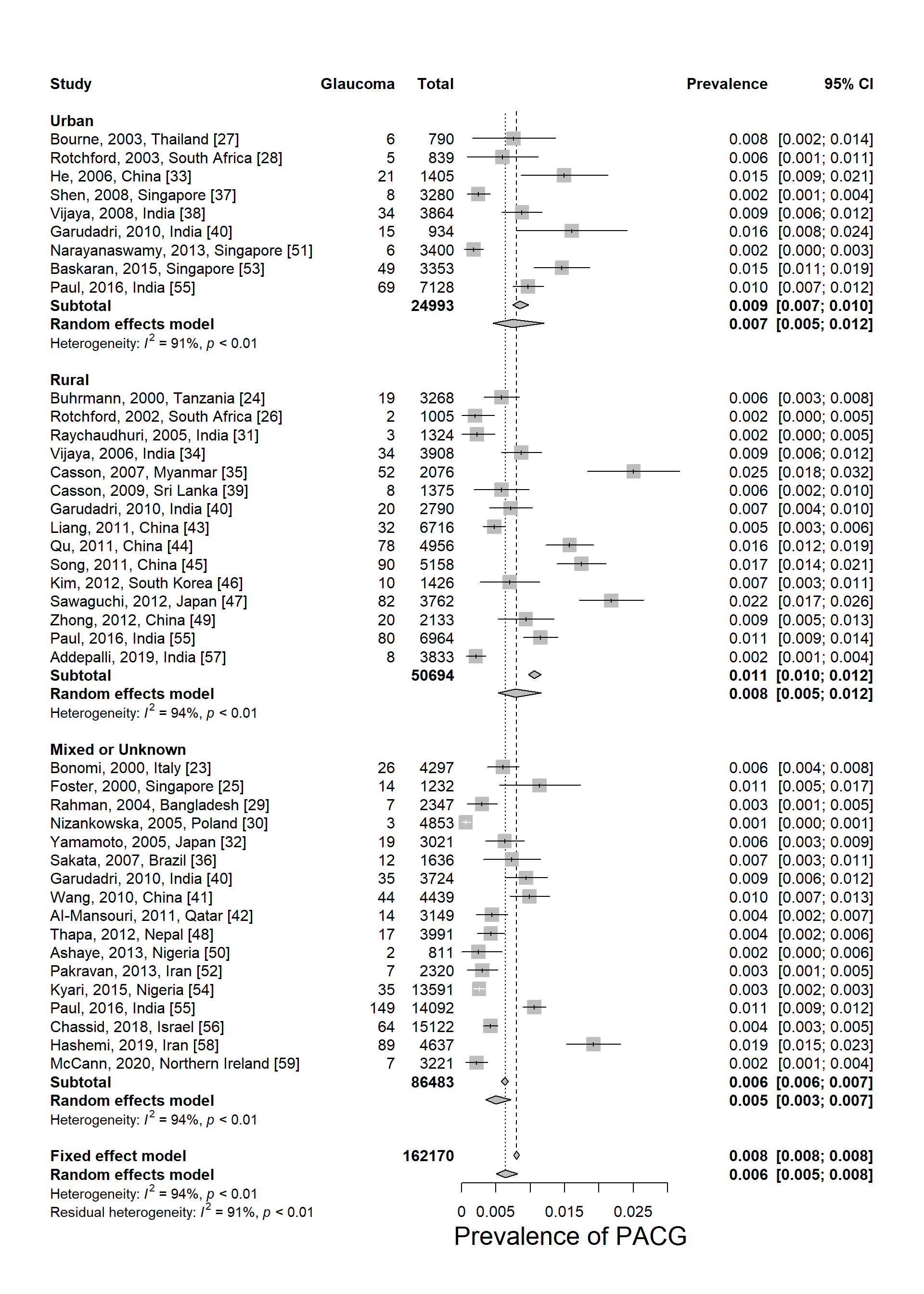
Supplementary Figure 8. PACG prevalence by habitation areas.
